# Supplementary material for: Tracking the genome-wide occupancy of Arabidopsis LEAFY COTYLEDON1 in endosperm development
Source: Commun Biol. 2025 May 21;8:771. doi: 10.1038/s42003-025-08075-y (PMC12095474; doi:10.1038/s42003-025-08075-y)
Supplement: Supplementary file 1 — Supplementary information [file 42003_2025_8075_MOESM1_ESM.pdf]

Tracking the genome-wide occupancy of Arabidopsis LEAFY COTYLEDON1 in endosperm development

Jingpu Song, Xin Xie, Ioannis Mavraganis, Bianyun Yu, Wenyun Shen, Hui Yang, Daoquan Xiang, Yangdou Wei, Yuhai Cui & Jitao Zou

Supplementary Figure 1

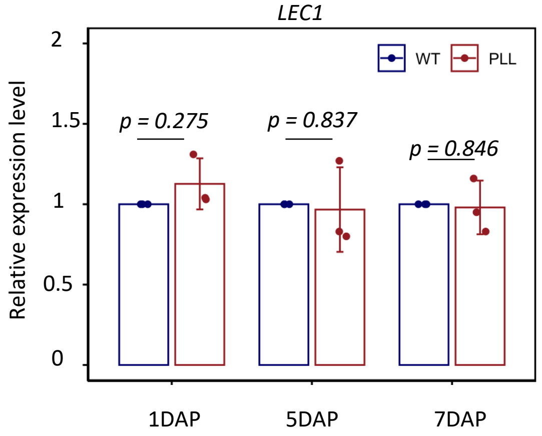

Supplementary Figure 1. Restoration of LEC1 expression in the PLL transgenic line. Relative expression of *LEC1* in the WT and PLL seeds at 1, 5, and 7 DAPs. The *CACS* gene was used as an internal control. Values are mean ± standard error of three biological replicates. *p* values were determined by conducting Student's t-test.

Supplementary Figure 2

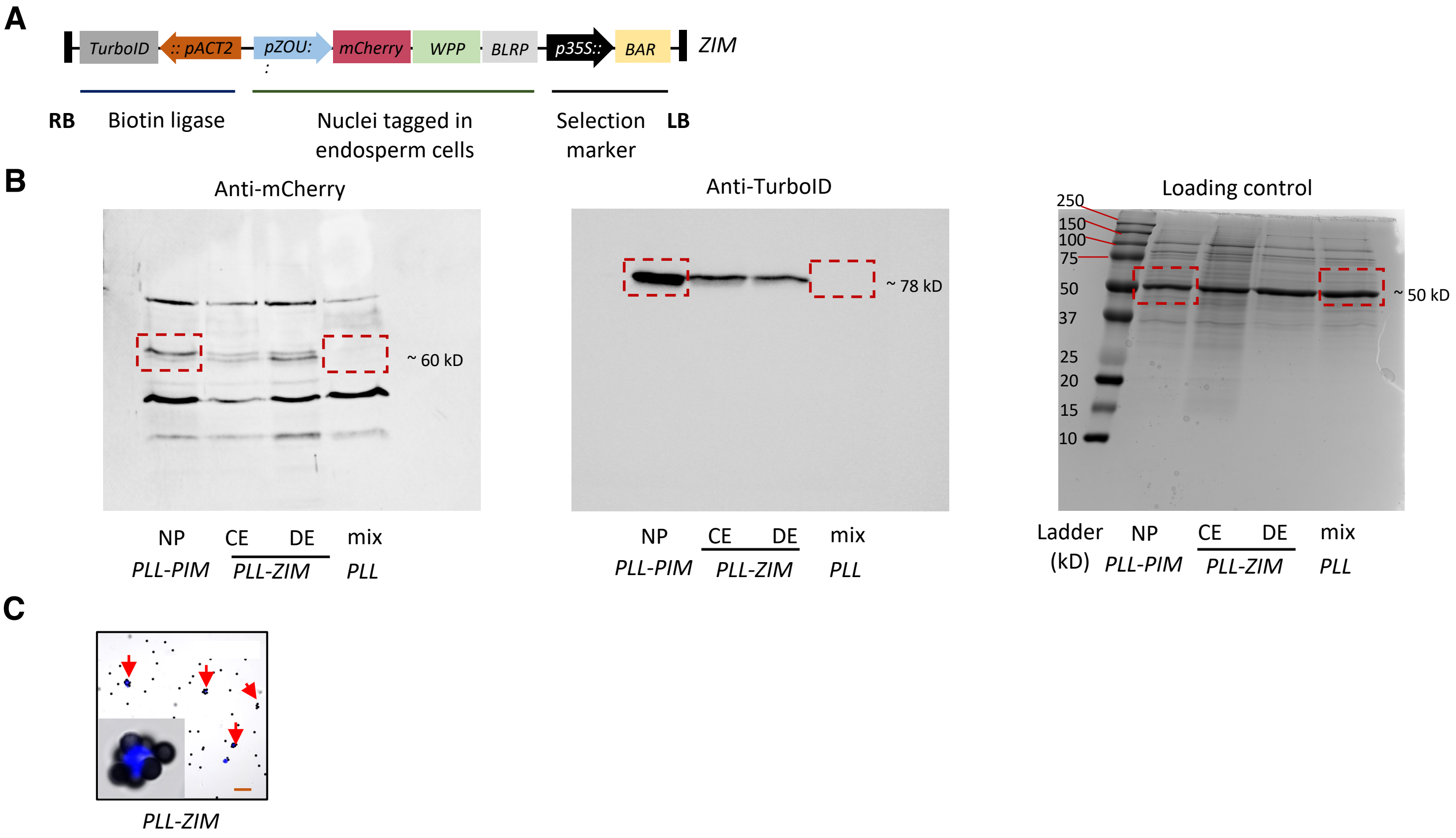

Supplementary Figure 2. Modification of INTACT system for cellular endosperm nuclei isolation. (A) Schematic diagrams showing the transgene structure of modified INTACT construct ZIM used for plant transformation. (B) Immunoblot (Uncropped gel images)showing the signals of mCherry (two bands, upper: biotinylated mCherry-WPP-BLRP lower band: non-biotinylated protein) and TurboID proteins in the developing seeds of the transgenic lines, PLL-PIM and PLL-ZIM. PLL seeds were used as negative control. Blot bands outlined in red boxes were shown in Fig. 1C. The images are representative of three independent replicates. NP, nuclei proliferation; CE, cellularization, DE, degeneration (C) Binding assay of beads-bound nuclei and free beads from PLL-ZIM developing seeds. Red arrows indicate beads-bound nuclei. Insets: magnified individual nuclei binding beads. Scale bar: 200 µm.

Supplementary Figure 3

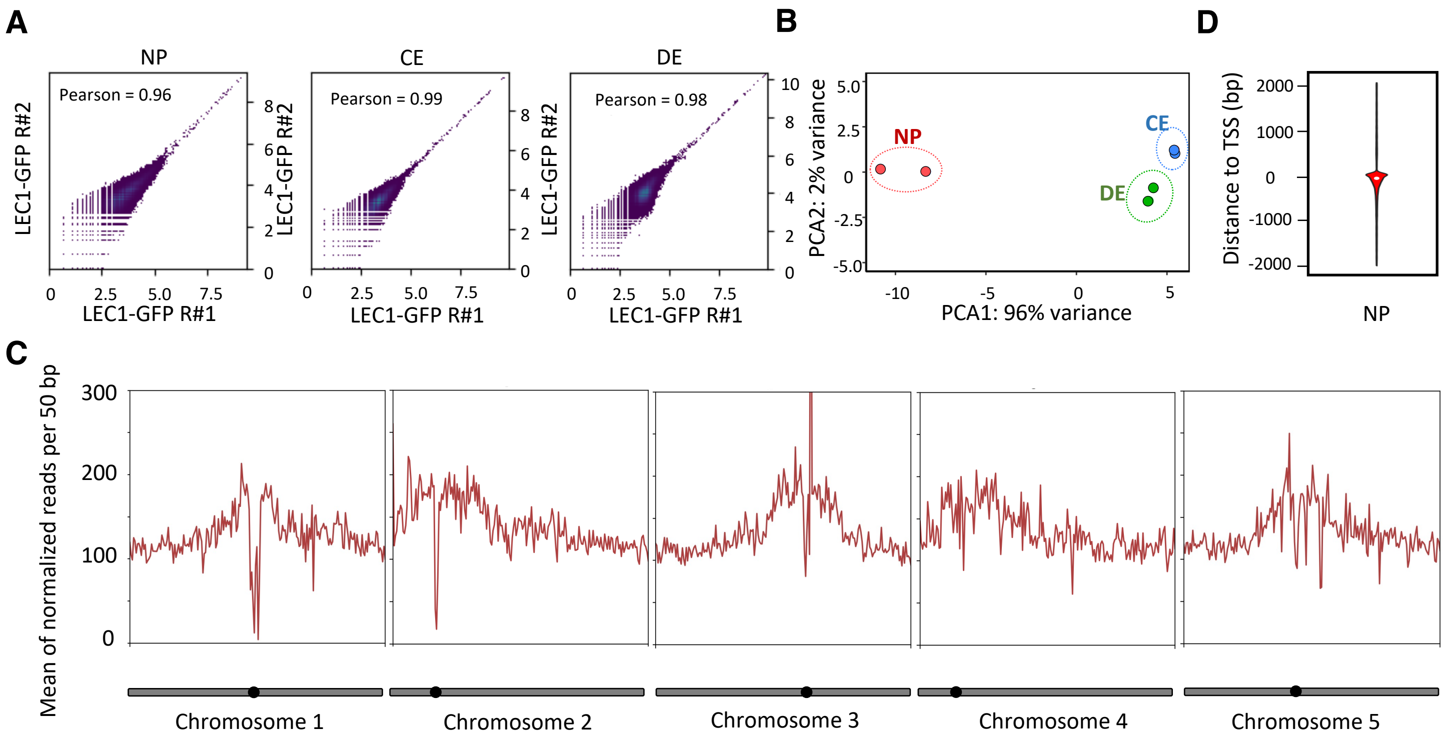

Supplementary Figure 3. The correlation analysis of the biological replicates of LEC1-GFP ChIP-Seq datasets. (A) Scatter plots showing high correlation between two biological replicates in ChIP-Seq at NP, CE, and DE, separately. (B) Principal component analysis (PCA) results indicates that the ChIP-Seq signal density profiles are separated by endosperm developmental stages. (C) Profile plots demonstrate that the distribution of LOGS in Arabidopsis chromosomes. Grey bars represent chromosome bodies and black dots indicate the centromeres. (D) Violine plots showing the distance of annotated ChIP-Seq peaks to the TSS at NP.

Supplementary Figure 4

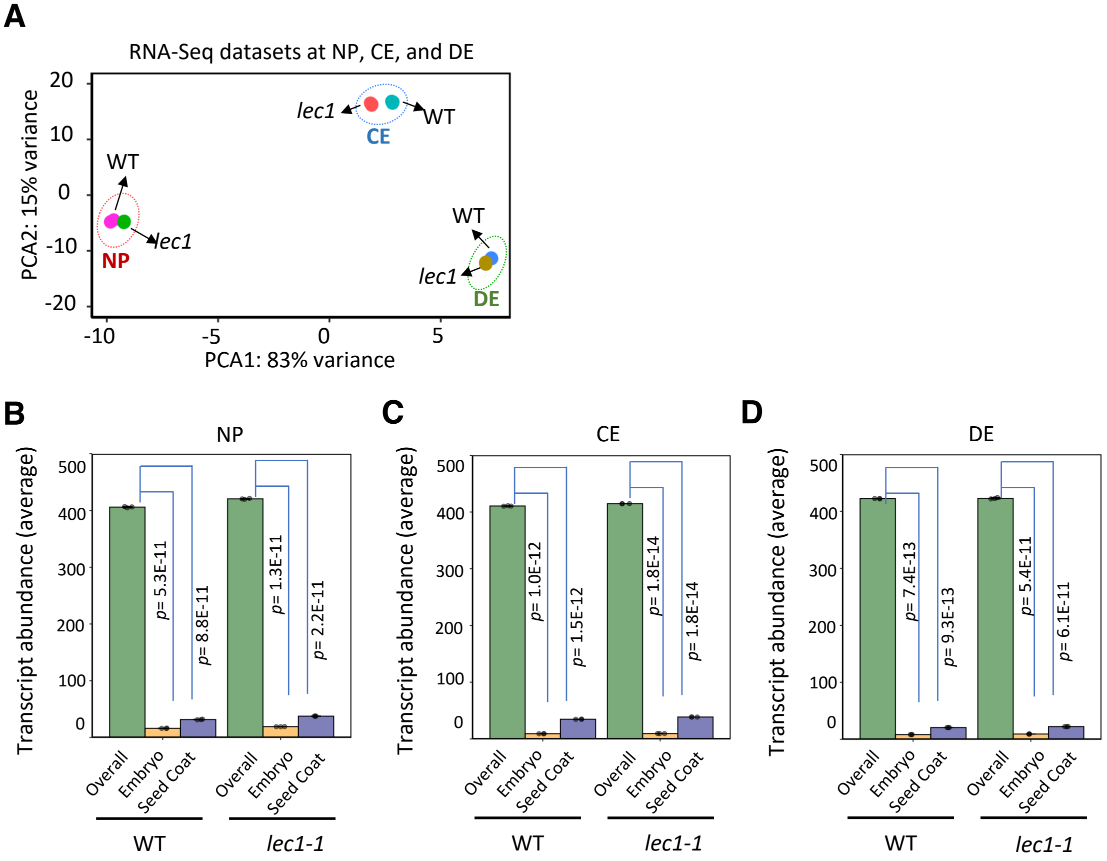

Supplementary Figure 4. Quality analysis of RNA-seq datasets. (A) Principal component analysis (PCA) results of RNA-Seq datasets indicates that the RNA-Seq signal density profiles are separated by endosperm developmental stages. (B-D) Average of gene transcripts of embryo- and seed coat-specific genes in the generated transcriptome profiles at NP, CE, and DE. The average transcript numbers of total genes (overall) at different genotypes and stages were included in the analysis. The list of embryo- and seed coat-specific genes were retrieved from previous published data (Pelletier, et al. 2017). Values are mean ± standard error of three biological replicates. *p* values were determined with one-way ANOVA followed by the post-hoc Tukey multiple comparison tests.

Supplementary Figure 5

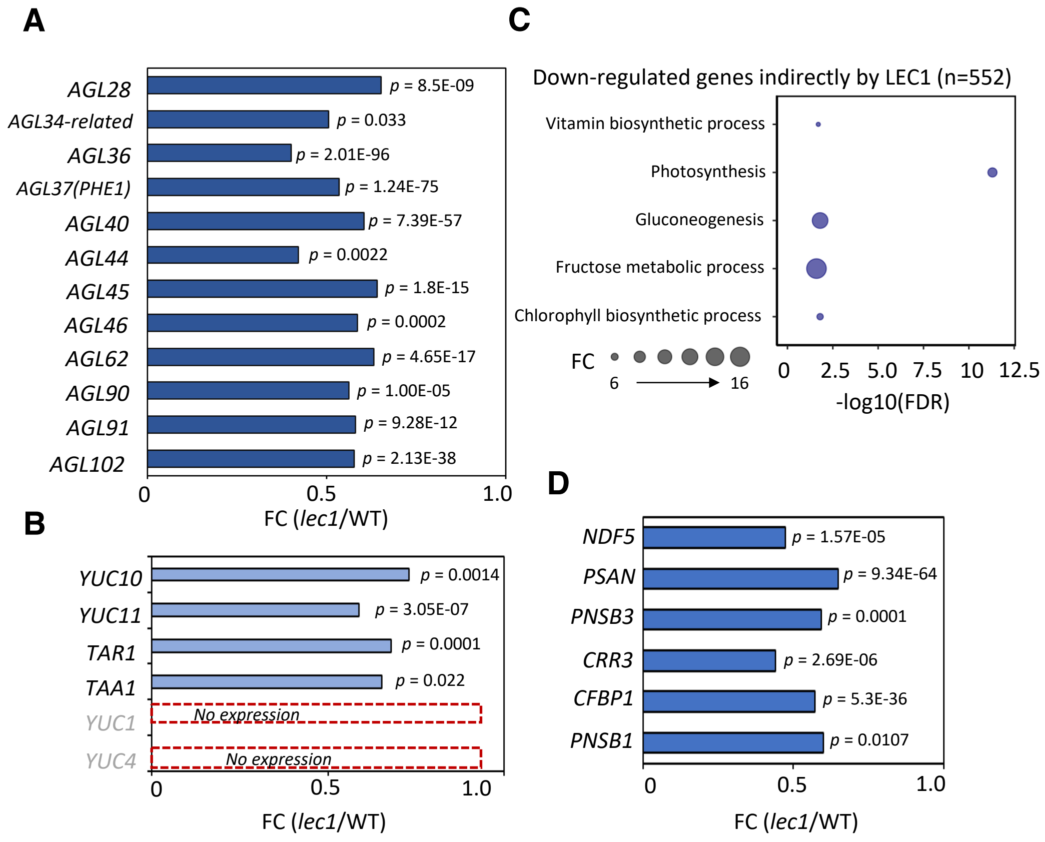

Supplementary Figure 5. Gene expression analysis of LEC1 directly and indirectly down-regulated targets in endosperm at NP. (A) Gene expression of LEC1-occupied down-regulated AGLs in the *lec1* endosperm at NP. (B) Gene expression levels of endosperm-specific genes involved in auxin biosynthesis. (C) GO analysis results elucidating the over-represented biological process (highlighted in navy blue colour) enriched among the non-LEC1-occupied down-regulated genes in *lec1* endosperm at NP. (D) Gene expression levels of down-regulated genes in photosynthesis.

Supplementary Figure 6

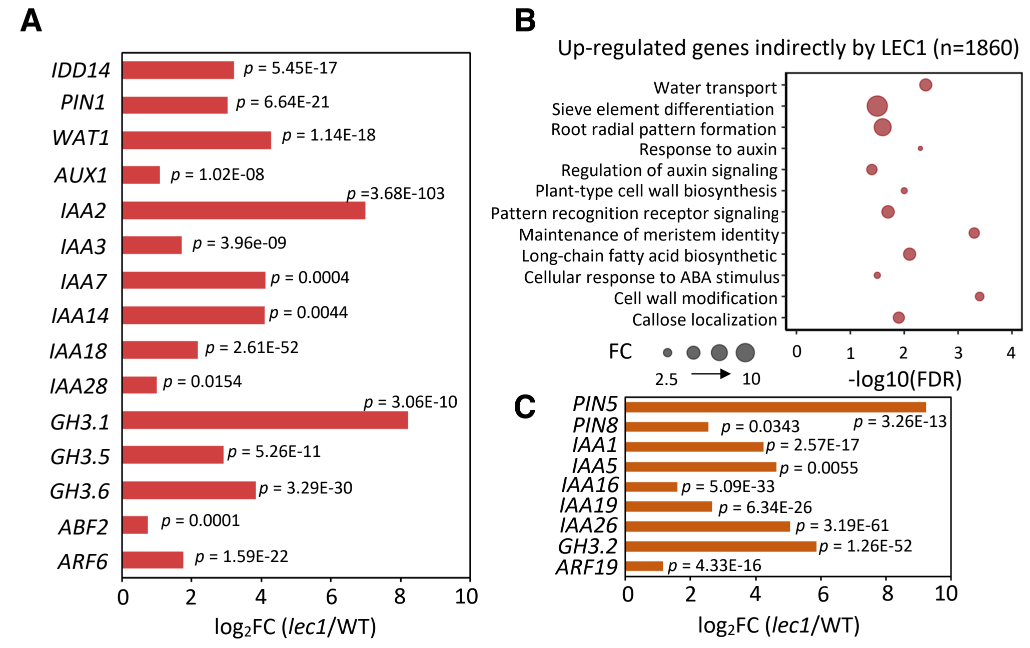

Supplementary Figure 6. Gene expression analysis of LEC1 directly and indirectly up-regulated targets in endosperm at NP. (A) Gene expression of LEC1-occupied up-regulated genes involved in auxin transportation and signaling in the *lec1* endosperm at NP. (B) GO analysis results elucidating the over-represented biological process (highlighted in red colour) enriched among the non-LEC1-occupied up-regulated genes in *lec1* endosperm at NP. (C) Gene expression of non-LEC1-occupied up-regulated genes involved in auxin response and signaling in the *lec1* endosperm at NP.

Supplementary Figure 7

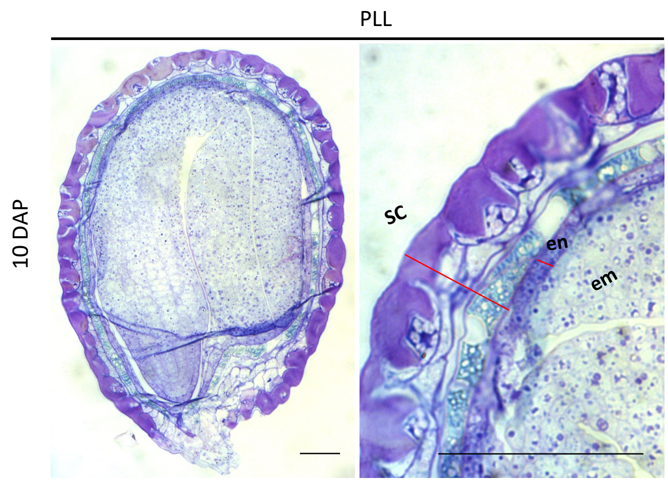

Supplementary Figure 7. Semi-thin section of PLL seed at 10 DAP. 10 DAP seeds collected from PLL plants were fixed and embedded for semi-thin section. SC, seed coat; en, endosperm; em, embryo; scale bars: 50 μm.

Supplementary Figure 8

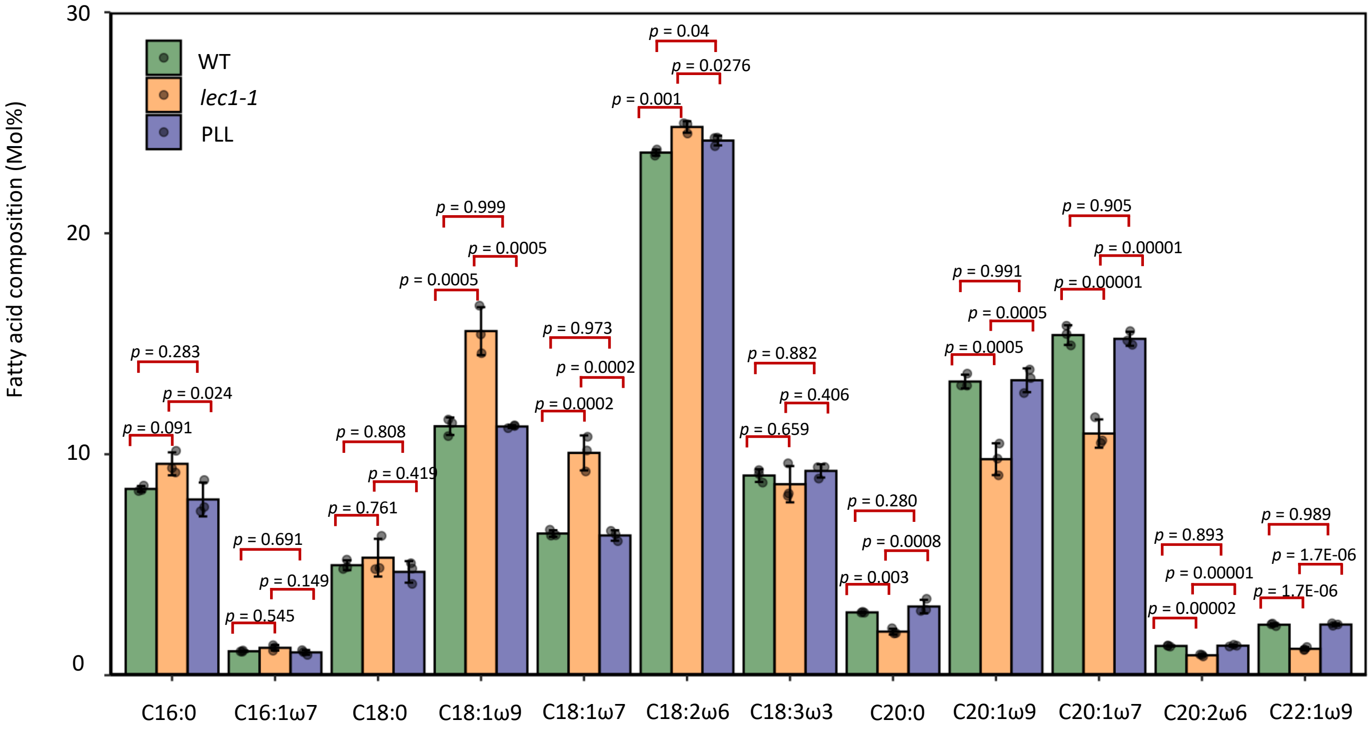

Supplementary Figure 8. Fatty acid composition analysis of endosperm dissected from mature seeds. Fatty acid composition profiles in endosperms isolated from WT, *lec1-1*, and PLL. Three independent replicates were conducted to calculate the means of values in each figure. Values are mean ± standard error of three biological replicates. *p* values were determined with one-way ANOVA followed by the post-hoc Tukey multiple comparison tests. For each replicate, endosperm tissue dissected from 15 seeds were pooled together for GC analysis.

Supplementary Figure 9

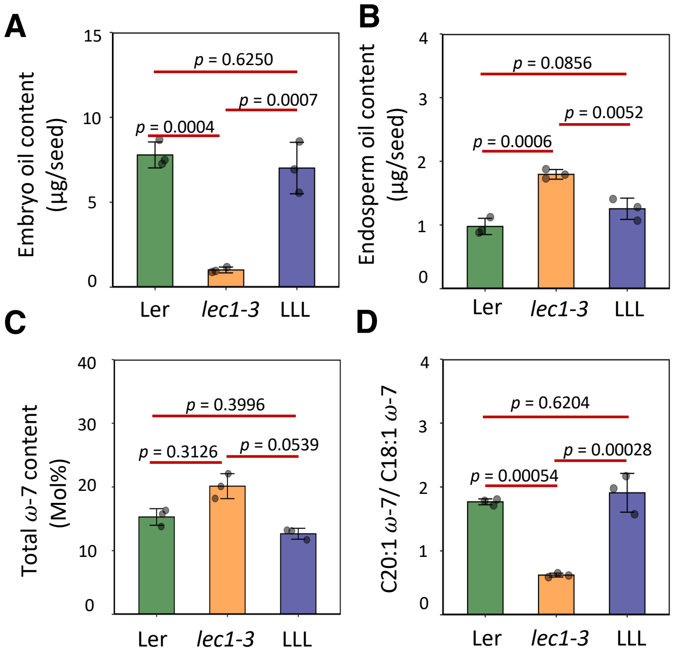

Supplementary Figure 9. Lack of LEC1 alters endosperm fatty acid composition in Ler. (A-D) Bargraphs showing the total oil content per seed in the embryo (A) and in the endosperm (B). (C) Total cis-ω-7 content accumulated in the endosperms of Ler (WT), *lec1-3*, and PLL seeds. (D) Statistic analysis of the ratios of C20:1 ω-7 versus C18:1 ω-7 in the endosperms of Ler, *lec1-3*, and LLL seeds. (A-D) Three independent replicates were conducted to calculate the means of values in each figure. For each replicate, 15 seeds of each genotype were dissected to separate embryo and endosperm tissues. Values are mean ± standard error of three biological replicates. *p* values were determined with one-way ANOVA followed by the post-hoc Tukey multiple comparison tests.

Supplementary Figure 10

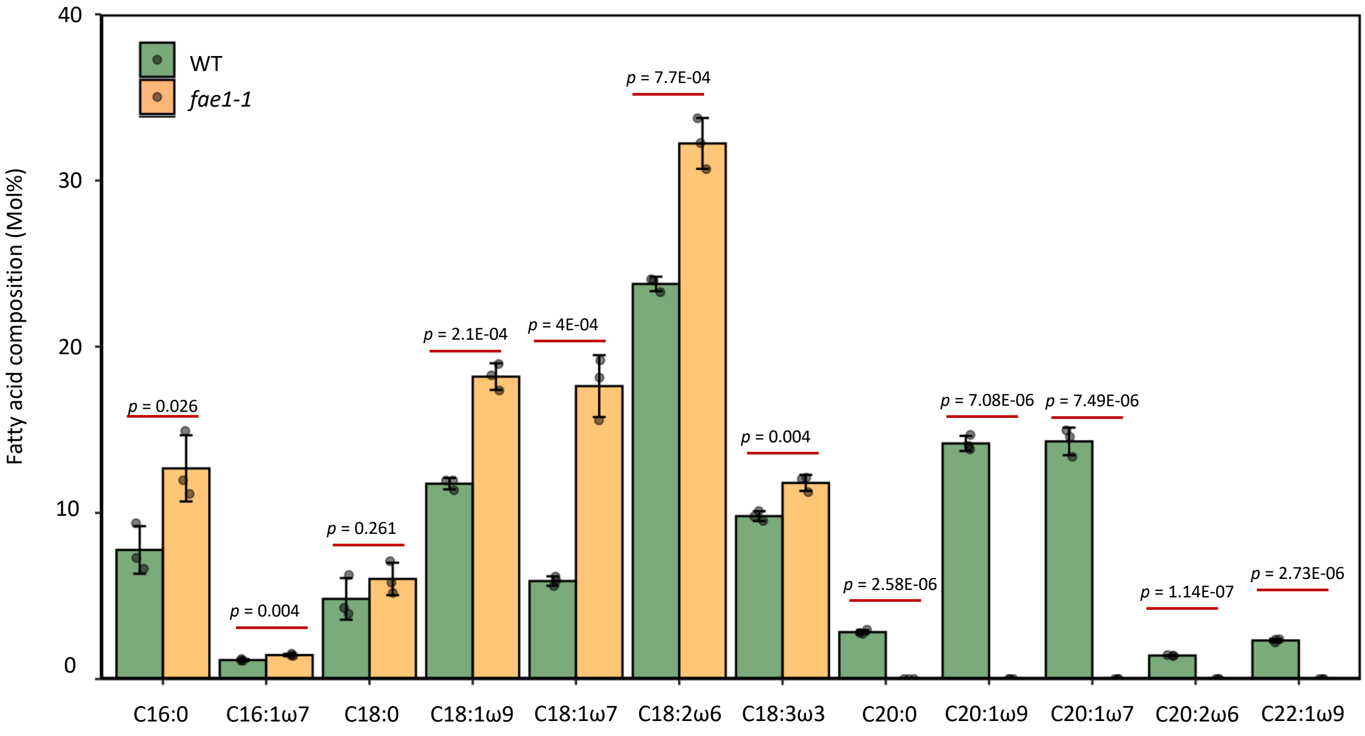

Supplementary Figure 10. Fatty acid composition profiles of endosperm dissected from mature WT and *fae1-1* seeds. Three independent replicates were conducted to calculate the means of values in each figure. Values are mean ± standard error of three biological replicates. For each replicate, endosperm tissue dissected from 15 seeds were pooled together for GC analysis. *p* values were determined by conducting Student's t-test.

Supplementary Figure 11

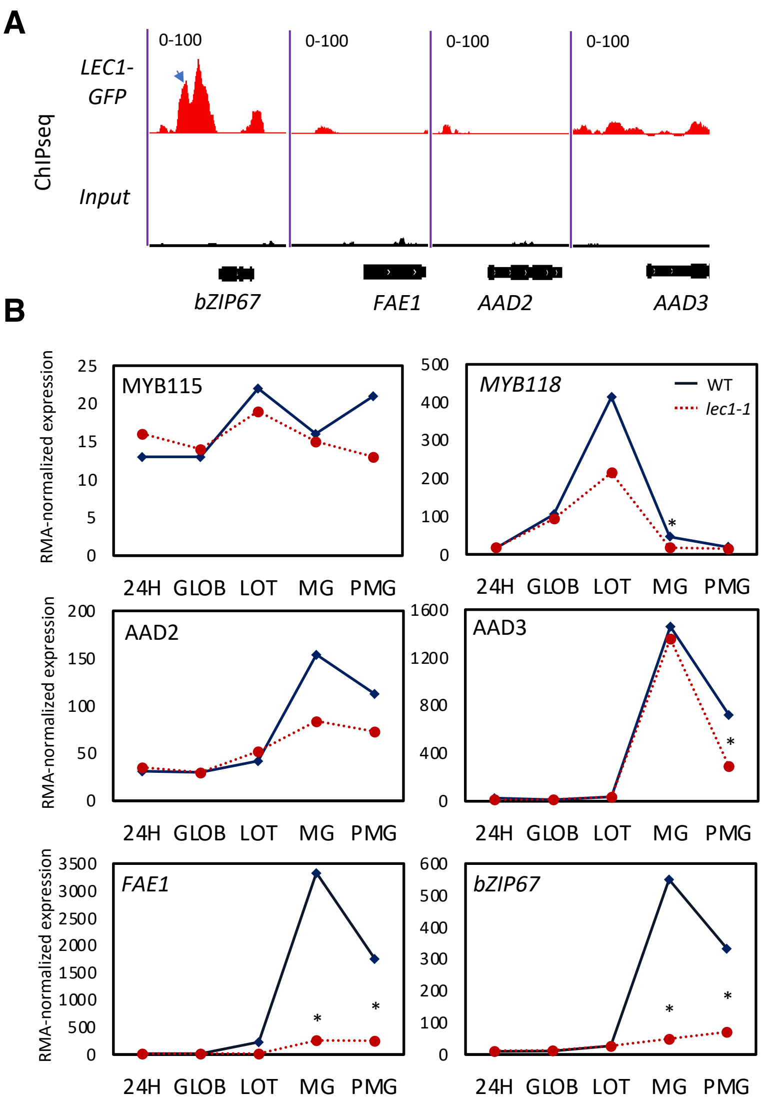

Supplementary Figure 11. LEC1 regulates genes responsible for cis-ω-7 fatty acid accumulation and elongation. (A) IGV views of ChIP-Seq signals indicate LEC1-occupied sites (pointed by arrowhead) on the promoter region of *bZIP67*, not on that of either *FAE1*, or *AAD2*, or *AAD3*. (B) Line graphs showing the transcription levels of *MYB115*, *MYB118*, *AAD2*, *AAD3*, *FAE1*, and *bZIP67* in WT and *lec1-1* seeds at different seed developmental stages (data collected from public resource). 24H, 24 hr after pollination; GLOB, globular stage; LOT, liner stage; MG, mature green; PMG, post mature green.

Supplementary Table 1. ChIP-Seq reads, mapping rates and peak calling information

Sample name	No. of sequenced reads	% of mapped reads	No. of called ChIP-seq peaks	ChIP-seq peaks present in both replicates and averaged ratio (IDR < 0.05)
NP-PLL-PIM-GFP-ChIP R#1	33,715,832	88.06	14,806	8,517
NP-PLL-PIM-GFP-ChIP R#2	37,750,795	91.33	16,431	
NP-PLL-PIM-input	28,905,178	96.74	/	
CE-PLL-ZIM-GFP-ChIP R#1	19,115,452	79.76	6,611	1,721
CE-PLL-ZIM-GFP-ChIP R#2	18,998,609	79.74	6,643	
CE-PLL-ZIM-input	24,979,345	97.65	/	
DE-PLL-ZIM-GFP-ChIP R#1	36,528,043	88.12	7,118	1,724
DE-PLL-ZIM-GFP-ChIP R#2	29,412,997	83.15	4,112	
DE-PLL-ZIM-input	34,291,183	98.01	/	

Supplementary Table 2. RNA-Seq reads and mapping information

Sample name	Total reads	Uniquely mapped reads	Mapping ratio
NP-WT-R#1	29036288	20394012	70.24%
NP-WT-R#2	32215289	23066317	71.60%
NP-WT-R#3	28918349	20756300	71.78%
NP-*lec1*-R#1	33679743	24047481	71.40%
NP-*lec1*-R#2	25888684	18764604	72.48%
NP-*lec1*-R#3	27424942	19557993	71.31%
CE-WT-R#1	24434385	18718255	76.61%
CE-WT-R#2	26387863	20379305	77.23%
CE-WT-R#3	22297316	18322235	74.10%
CE-*lec1*-R#1	22235249	17138789	77.08%
CE-*lec1*-R#2	23013113	17813006	77.40%
CE-*lec1*-R#3	28961587	22424085	77.43%
DE-WT-R#1	29856278	23206002	77.73%
DE-WT-R#2	23832611	18569483	77.92%
DE-WT-R#3	29368224	22808009	77.66%
DE-*lec1*-R#1	24966484	19264921	77.16%
DE-*lec1*-R#2	19839904	15119354	76.21%
DE-*lec1*-R#3	26577683	20331626	76.50%

Supplementary Table 3. Primers used for this study

Experiment	Primer ID	Sequence
pPHE1::mCherry-NLS	PAC1-pPHE1-F	CTTAATTAAACTGTTGATCCGGTGAATATCC
	AVRII-pPHE1-R	TCACCTAGGATCTCTTATCTTTTTCTTTTGTGTATTTTG
	Pac1-AvrII-mcherry-F	CTTAATTAACCTAGGATGGTGAGCAAGGGCGAGG
	Asc1-NLS-mcherry-R	TGGCGCGCCTCAGTCCAACTTGACCCTCTTGGCAGCAGGCTTGTACAGCTCGTCCATGCC
INTACT component construction	Pme1-TurboID	GGGTTTAAACTATGATCTCAAATACATTGATACATATC
	Asc1-TurBOID-Nos	TTGGCGCGCCGAATTCTCATGTTTGACAGC
	Pac1-AvrII-RAN-F	CTTAATTAACCTAGGATGGATCATTCAGCGAAAAC
	Spe1-NOS-R	CCACTAGTGAATTCTCATGTTTGACAGC
	Pac1-mCherry-F	CTTAATTAAATGGTGAGCAAGGGCGAGG
	AvrII-mCherry-R	TTCCTAGGCTTGTACAGCTCGTCCATGCC
promoter pPHE1 for PIM	BP-pPHE1-F	GGGGACAAGTTTGTACAAAAAAGCAGGCTACTGTTGATCCGGTGAATATCC
	BP-pPHE1-R	GGGGACCACTTTGTACAAGAAAGCTGGGTCATCTCTTATCTTTTTCTTTTGTGTATTTTG
promoter pZOU for ZIM	BP-pZOU-F	GGGGACAAGTTTGTACAAAAAAGCAGGCTTACCACCCTATACTTATTAGACAG
	BP-pZOU-R	GGGGACCACTTTGTACAAGAAAGCTGGGTCATTGAATTGAATGCTCATTTTACC
ChIP-qPCR	FIE-CHIPqPCR-F	TGATTGATACGATCGAAGTTT
	FIE-CHIPqPCR-R	ACGTGATACCATTTAAATCCA
	YUC10-CHIPqPCR-F	GTTTTGTCGGAAATAAAACA
	YUC10-CHIPqPCR-R	TGGAAAATGATCCATAGTTG
	VIM1-CHIPqPCR-F	ATTCGGGATTAAAATTGTTC
	VIM1-CHIPqPCR-R	TTGCAAATCGATATAAACCA
	VIM2-CHIPqPCR-F	ATTGCCGATTAACTAACACG
	VIM2-CHIPqPCR-R	TTGCTACCAAAATTTCAATG
	AGL61-CHIPqPCR-F	TTTGATTTGCAACCTCTTTT
	AGL61-CHIPqPCR-R	GGTTTTGTCTTTAATTTTGTGG
	BZIP62-CHIPqPCR-F	GAGAGAGCGACACGTGTAAT
	BZIP62-CHIPqPCR-R	TGCTGACTTGGTAACAAAAA
RT-qPCR	CACS-qF	ACTCAGGAAGGTGTACGGTCA
	CACS-qR	TGCATTTGGAACAGGTTTGT
	LEC1-qF	CGATCGTGGTTCTGCACTTA
	LEC1-qR	ATTCATCTTGACCCGACGAC
