# Supplementary material for: Tracking the genome-wide occupancy of Arabidopsis LEAFY COTYLEDON1 in endosperm development
Source: Commun Biol. 2025 May 21;8:771. doi: 10.1038/s42003-025-08075-y (PMC12095474; doi:10.1038/s42003-025-08075-y)
Supplement: Supplementary file 2 — Description of additional supplementary materials [file 42003_2025_8075_MOESM2_ESM.pdf]

Description of Additional Supplementary Files

File name: Supplementary Data 1

Description: List of LEC1-occupied genes in developing endosperm at nuclei proliferation (NP)

File name: Supplementary Data 2

Description: DEGs in lec1 mutant endosperm at NP, CE, and DE

File name: Supplementary Data 3

Description: LEC1-targets in endosperm at different developmental stages

File name: Supplementary Data 4

Description: comparison of LEC1-targets in embryo and endosperm

File name: Supplementary Data 5

Description: Source file
