# Supplementary material for: Tracking the genome-wide occupancy of Arabidopsis LEAFY COTYLEDON1 in endosperm development
Source: Commun Biol. 2025 May 21;8:771. doi: 10.1038/s42003-025-08075-y (PMC12095474; doi:10.1038/s42003-025-08075-y)
Supplement: Supplementary file 8 — Reporting summary [file 42003_2025_8075_MOESM8_ESM.pdf]

nature portfolio

Corresponding author(s): Jitao Zou, Yuhai Cui

Last updated by author(s): Apr 8, 2025

Reporting Summary

Nature Portfolio wishes to improve the reproducibility of the work that we publish. This form provides structure for consistency and transparency in reporting. For further information on Nature Portfolio policies, see our Editorial Policies and the Editorial Policy Checklist.

Statistics

For all statistical analyses, confirm that the following items are present in the figure legend, table legend, main text, or Methods section.

n/a	Confirmed	
☐	☒	The exact sample size (*n*) for each experimental group/condition, given as a discrete number and unit of measurement
☐	☒	A statement on whether measurements were taken from distinct samples or whether the same sample was measured repeatedly
☐	☒	The statistical test(s) used AND whether they are one- or two-sided<br>*Only common tests should be described solely by name; describe more complex techniques in the Methods section.*
☒	☐	A description of all covariates tested
☒	☐	A description of any assumptions or corrections, such as tests of normality and adjustment for multiple comparisons
☐	☒	A full description of the statistical parameters including central tendency (e.g. means) or other basic estimates (e.g. regression coefficient) AND variation (e.g. standard deviation) or associated estimates of uncertainty (e.g. confidence intervals)
☐	☒	For null hypothesis testing, the test statistic (e.g. *F*, *t*, *r*) with confidence intervals, effect sizes, degrees of freedom and *P* value noted<br>*Give P values as exact values whenever suitable.*
☒	☐	For Bayesian analysis, information on the choice of priors and Markov chain Monte Carlo settings
☒	☐	For hierarchical and complex designs, identification of the appropriate level for tests and full reporting of outcomes
☐	☒	Estimates of effect sizes (e.g. Cohen's *d*, Pearson's *r*), indicating how they were calculated

Our web collection on statistics for biologists contains articles on many of the points above.

Software and code

Policy information about availability of computer code

Data collection	Confocal images were analyzed using the imaging software: LSM Image Browser and Fiji ImageJ software. ChIP-seq and RNA-seq datasets were collected using Illumina NOVAseq 6000.
Data analysis	R v.4.1.2 (https://www.r-project.org) was used for the statistical analyses. Student's t-tests and One-way ANNOVA analyses and post-hoc Tukey tests were conducted to determine the significant difference. Boxplot graphs were generated using R studio.

For manuscripts utilizing custom algorithms or software that are central to the research but not yet described in published literature, software must be made available to editors and reviewers. We strongly encourage code deposition in a community repository (e.g. GitHub). See the Nature Portfolio guidelines for submitting code & software for further information.

Data

Policy information about availability of data

All manuscripts must include a data availability statement. This statement should provide the following information, where applicable:

- Accession codes, unique identifiers, or web links for publicly available datasets
- A description of any restrictions on data availability
- For clinical datasets or third party data, please ensure that the statement adheres to our policy

All ChIP-Seq and RNA-Seq raw datasets and process data files generated from this study were deposited into the Nation Center for Biotechnology Information (NCBI) under project ID PRJNA1100724.

Research involving human participants, their data, or biological material

Policy information about studies with human participants or human data. See also policy information about sex, gender (identity/presentation), and sexual orientation and race, ethnicity and racism.

Reporting on sex and gender	NA
Reporting on race, ethnicity, or other socially relevant groupings	NA
Population characteristics	NA
Recruitment	NA
Ethics oversight	NA

Note that full information on the approval of the study protocol must also be provided in the manuscript.

Field-specific reporting

Please select the one below that is the best fit for your research. If you are not sure, read the appropriate sections before making your selection.

☒ Life sciences ☐ Behavioural & social sciences ☐ Ecological, evolutionary & environmental sciences

For a reference copy of the document with all sections, see nature.com/documents/nr-reporting-summary-flat.pdf

Life sciences study design

All studies must disclose on these points even when the disclosure is negative.

Sample size	Sample sizes were estimated based on our experiences in performing similar experiments and published works. For ChIP-Seq, usually two independent biological replicates for endosperm stage. For RNA-seq and qPCR, usually three biological replicates were performed in majority published papers. Therefore, we performed three independent biological replicates for each genotype. The exact sample size for each experiment was described clearly in the figure legends.
Data exclusions	No data were excluded from the analysis
Replication	All attempts at replication were successful.
Randomization	All samples were allocated randomly into experimental groups. All the biological replicates were grown and collected independently at different times, although all the replicates were extracted and processed at the same time.
Blinding	For biological replicates, independent researchers (not related to this project)were asked to collect plant samples, perform experiments, and conduct data analysis.

Reporting for specific materials, systems and methods

We require information from authors about some types of materials, experimental systems and methods used in many studies. Here, indicate whether each material, system or method listed is relevant to your study. If you are not sure if a list item applies to your research, read the appropriate section before selecting a response.

Materials & experimental systems

n/a	Involved in the study	
☐	☒	Antibodies
☒	☐	Eukaryotic cell lines
☒	☐	Palaeontology and archaeology
☒	☐	Animals and other organisms
☒	☐	Clinical data
☒	☐	Dual use research of concern
☐	☒	Plants

Methods

n/a	Involved in the study	
☐	☒	ChIP-seq
☒	☐	Flow cytometry
☒	☐	MRI-based neuroimaging

Antibodies

Antibodies used	Anti-GFP (Abcam, Cambridge, UK, Cat#ab290) ;Anti-TurboID (Agrisera, Sweden, Product no. AS204440) ; Anti-mcherry (Abcam, Cambridge, UK, Cat#AB167453); Rabbit IgG (Abcam, Cambridge, UK, Cat#ab171870)
Validation	Negative controls were introduced in Western blot assays for antibody validation. Validations for all primary antibodies used in this study are avaliable online.

Dual use research of concern

Policy information about dual use research of concern

Hazards

Could the accidental, deliberate or reckless misuse of agents or technologies generated in the work, or the application of information presented in the manuscript, pose a threat to:

No	Yes	
☒	☐	Public health
☒	☐	National security
☒	☐	Crops and/or livestock
☒	☐	Ecosystems
☒	☐	Any other significant area

Experiments of concern

Does the work involve any of these experiments of concern:

No	Yes	
☒	☐	Demonstrate how to render a vaccine ineffective
☒	☐	Confer resistance to therapeutically useful antibiotics or antiviral agents
☒	☐	Enhance the virulence of a pathogen or render a nonpathogen virulent
☒	☐	Increase transmissibility of a pathogen
☒	☐	Alter the host range of a pathogen
☒	☐	Enable evasion of diagnostic/detection modalities
☒	☐	Enable the weaponization of a biological agent or toxin
☒	☐	Any other potentially harmful combination of experiments and agents

Plants

Seed stocks	Arabidopsis Biological Resources Center (lec1-1, SALK_131219; lec1-3, CS5739)
Novel plant genotypes	Plant transformation experiments were conducted by following floral dipping method
Authentication	Mutant lines have been genotyped in previous research.

ChIP-seq

Data deposition

☒ Confirm that both raw and final processed data have been deposited in a public database such as GEO.

☒ Confirm that you have deposited or provided access to graph files (e.g. BED files) for the called peaks.

Data access links<br>*May remain private before publication.*	https://www.ncbi.nlm.nih.gov/geo/query/acc.cgi?acc=GSE291311

Files in database submission	LEC1_GFP_NP1.bed; LEC1_GFP_NP2.bed; LEC1_GFP_CE1.bed; LEC1_GFP_CE2.bed; LEC1_GFP_DE1.bed; LEC1_GFP_DE2.bed;
Genome browser session (e.g. UCSC)	http://genome.ucsc.edu/

Methodology

Replicates	Two independent biological replicates for each stage
Sequencing depth	The average reads for each sample are around 30 million, pair-end, Unique mapping rate is approximately 90%.
Antibodies	Anti-GFP (Abcam, Cambridge, UK, Cat#ab290)
Peak calling parameters	Peak calling was conducted using MACS2 (Galaxy v 2.2.7.1) with default parameters
Data quality	The information is available in Extended Table 1
Software	Raw reads were uploaded to Galaxy (http://usegalaxy.org/). Sequences were trimmed with Trim Galore (Galaxy v 0.6.7)40 before mapping to the Arabidopsis reference genome (TAIR10) with Bowtie241 for Illumina (Galaxy v 2.5.0) for DNA sequencing with pair end and other settings as default. Unmapped and PCR duplicates were filtered out using SAMtools42, only uniquely and perfectly mapped reads were retained for further analysis. The bigwig format files were generated by bamCoverage with “bin size 10” and “normalize to RPKM (reads per kilobase per million)” in Deeptools43 for visualization using Integrative Genomics Viewer44. Peak calling was conducted using MACS2 (Galaxy v 2.2.7.1) with default parameters45. Sequencing reads from Input-DNA were used as controls. Common peaks shared by two biological replicates were determined by IDR (Galaxy v 2.0.3) with values less than 0.05 for further analysis46. The ChIPseeker(Galaxy v 1.18.0) was used to assign peaks to proximal genes.
